# Concurrent validity of an immersive virtual reality version of the *Box and Block Test* to assess manual dexterity among patients with stroke

**DOI:** 10.1186/s12984-022-00981-0

**Published:** 2022-01-22

**Authors:** Gauthier Everard, Yasmine Otmane-Tolba, Zélie Rosselli, Thomas Pellissier, Khawla Ajana, Stéphanie Dehem, Edouard Auvinet, Martin Gareth Edwards, Julien Lebleu, Thierry Lejeune

**Affiliations:** 1 Neuro Musculo Skeletal Lab (NMSK), Institut de Recherche Expérimentale et Clinique, Secteur des Sciences de la Santé, Université Catholique de Louvain, Brussels, Belgium; 2 ECAM- Institut Supérieur Industriel, Brussels, Belgium; 3 Psychological Sciences Research Institute (IPSY), Université Catholique de Louvain, Louvain-la-Neuve, Belgium; 4 Service de Médecine Physique et Réadaptation, Cliniques Universitaires Saint-Luc, Brussels, Belgium; 5 Louvain Bionics, Université Catholique de Louvain, Louvain-la-Neuve, Belgium; 6 Médecine Physique et Réadaptation, Cliniques Universitaires Saint-Luc, Avenue Hippocrate 10, 1200 Brussels, Belgium

**Keywords:** Stroke, Virtual reality, Assessment, Self-rehabilitation, Tele rehabilitation

## Abstract

**Background:**

After a stroke, experts recommend regular monitoring and kinematic assessments of patients to objectively measure motor recovery. With the rise of new technologies and increasing needs for neurorehabilitation, an interest in virtual reality has emerged. In this context, we have developed an immersive virtual reality version of the *Box and Block Test* (BBT-VR). The aim of this study was to assess the concurrent validity of the BBT-VR among patients with stroke and healthy participants.

**Methods:**

Twenty-three healthy participants and 22 patients with stroke were asked to perform the classical Box and Block Test (BBT) and BBT-VR three times with both hands. Concurrent validity was assessed through correlations between these two tests and reliability of the BBT-VR through correlation on test–retest. Usability of the BBT-VR was also evaluated with the *System Usability Scale.* Hand kinematic data extracted from controller’s 3D position allowed to compute mean velocity (V_mean_), peak velocity (V_peak_) and smoothness (SPARC).

**Results:**

Results showed strong correlations between the number of blocks displaced with the BBT and the BBT-VR among patients with stroke for affected (r = 0.89; p < 0.001) and less-affected hands (r = 0.76; p < 0.001) and healthy participants for dominant (r = 0.58; p < 0.01) and non-dominant hands (r = 0.68; p < 0.001). Reliability for test–retest was excellent (ICC > 0.8; p < 0.001) and usability almost excellent (*System Usability Scale* = 79 ± 12.34%). On average participants moved between 30 and 40% less blocks during the BBT-VR than during the BBT. Healthy participants demonstrated significantly higher kinematic measures (V_mean_ = 0.22 ± 0.086 ms^−1^; V_peak_ = 0.96 ± 0.341 ms^−1^; SPARC = − 3.31 ± 0.862) than patients with stroke (V_mean_ = 0.12 ± 0.052 ms^−1^; V_peak_ = 0.60 ± 0.202 ms^−1^; SPARC = − 5.04[− 7.050 to − 3.682]).

**Conclusion:**

The BBT-VR is a usable, valid and reliable test to assess manual dexterity, providing kinematic parameters, in a population of patients with stroke and healthy participants.

*Trial registration *http://www.clinicaltrials.gov; Unique identifier: NCT04694833, Date of registration: 11/24/2020

**Supplementary Information:**

The online version contains supplementary material available at 10.1186/s12984-022-00981-0.

## Background

After a stroke, almost 80% of patients present with upper-limb impairments such as hemiparesis and sensory deficits [1]. These dysfunctions may be responsible for impaired manual dexterity which leads to activity limitation and participation restriction [2]. Most patients will progressively recover from these deficits, typically reaching a plateau of recovery after 6 months to one year of rehabilitation [3]. In clinical routine and research, it is recommended to frequently evaluate patients at different time points in order to establish prognostics and plan rehabilitation projects. In this context, experts recommend regular monitoring and assessment using validated measures [4]. Experts also recommend implementing kinematic analysis in the evaluation of patients to objectively measure recovery [5].

The *Box and Block Test* (BBT) is one of the most used and recommended tools to evaluate unilateral manual dexterity [6, 7]. The BBT comprises a box divided into two compartments by a wooden separation and 150 small wood cubic blocks (1-inch). The BBT consists of displacing a maximum number of small blocks, one-by-one, from the compartment located on the tested side to the other using the same hand (taking care to pass over the separation). The score is obtained by counting the number of blocks correctly displaced within 60 s. The BBT has shown good inter/intra-examinator reliability properties and has been validated in healthy participants and adults with stroke [7–12].

With the rise of stroke incidence, there are increasing needs for neurorehabilitation and for research to new effective treatments methods using immersive and non-immersive virtual reality (VR). VR provides an effective rehabilitation approach that offers the possibility to deliver goal-oriented tasks, multisensorial and performance feedback, and increased treatment intensity [13–16]. Several studies also suggested that VR could improve patients’ adherence by increasing motivation [17, 18]. The addition of playful interventions such as those provided by serious games may be an effective approach to increase adherence in patients with stroke [19]. Moreover, when provided with an autonomous headset, VR offers patients the possibility to rehabilitate alone and at home. Most VR systems can track hand movements using integrated infrared camera systems or using inertial measurements units present in the controllers. However, despite its potential, the use of immersive VR to perform functional assessments and to analyse upper limb kinematics has been so far under explored for post-stroke rehabilitation [20].

The interest of developing such virtual tests is multiple. It may help clinicians to objectively measure patient’s performance using the automatic computation of quantitative measures and therefore reduce inter-rater subjectivity present in the classical evaluation. It may also be part of a complete home-based virtual rehabilitation system where patients could self-rehabilitate through virtual serious games on one side, and, on the other, assess their cognitive and motor improvements using validated virtual evaluations. To respond to these needs and to the growing interest in telemedicine, we developed a non-commercial immersive virtual version of the BBT (BBT-VR).

In the literature, there are only three studies that have developed a virtual BBT. One study used a non-immersive VR device involving a depth-sensing camera [21]. Results showed good correlations between the BBT and the non-immersive BBT among patients with stroke. However, non-immersive VR does not enable tactile and realistic 3D visual feedback of participant’s hand position, reducing the user experience of the patient. Another published paper developed a virtual BBT using an immersive headset with hand tracking technology (*Leap Motion Controller—Ultraleap*) to assess unilateral manual dexterity. The study evaluated the concurrent validity of the virtual BBT among patients with Parkinson Disease [22] and results indicated moderate correlation between the virtual BBT and BBT scores. The third paper developed a virtual BBT using an immersive headset with hand-tracking technology [23]. Validity was not assessed, but results showed that healthy participants, on average, displaced 35 more blocks in the BBT than in the virtual BBT. In all three of these studies, the virtual BBT systems required the use of a powerful expensive computer making the tests less portable, and they did not enable tactile feedback for block interaction, which reduces the user experience. In addition, none of these studies provided kinematic measures.

This study here aimed to develop an immersive virtual version of the BBT (BBT-VR) and assess concurrent validity among patients with stroke and healthy participants. The hypothesis was that the BBT-VR and BBT scores obtained by participants would be correlated. Secondary objectives were to assess the short-term test–retest reliability and usability of the BBT-VR test, and to compute and compare hand kinematics analyses.

## Methods

### Study design

This multicentric concurrent validity study was approved by the Saint-Luc-UCLouvain Hospital-Faculty Ethics Committee (Belgium) and is registered on clinicaltrial.gov (NCT04694833). The data that support the findings of this study are available in Additional file 1.

### Participants

Patients with stroke and healthy participants were recruited using convenience sampling from the physical medicine and rehabilitation departments of the Cliniques universitaires Saint-Luc and Mont-Godinne University Hospital between November 2020 and March 2021. All participants provided written informed consent before enrolment. Patients were included if they were diagnosed with a first-ever stroke according to the World Health Organization criteria, with cerebral lesions confirmed by CT or magnetic resonance imaging, if they had an upper-limb hemiparesis and had normal or corrected-to-normal vision. Patients with risk of epilepsy or any neurological and orthopaedical condition other than stroke that could affect the upper extremity function were excluded. Healthy participants were included if they had normal or corrected-to-normal vision and were excluded if they presented with any neurological or orthopaedic disorder that could affect the upper-limb movement.

### Materials

The BBT-VR was developed using the *Oculus Quest 1* (*Facebook*) and combines the use of the haptic controllers and headset to assess unilateral manual dexterity. This BBT-VR enables to automatically calculate the score according to the number of virtual blocks correctly displaced by the tested hand, making it possible for patients to be evaluated in an autonomous manner. An additional movie file show this in more detail (see Additional file 2).

**Fig. 1:**
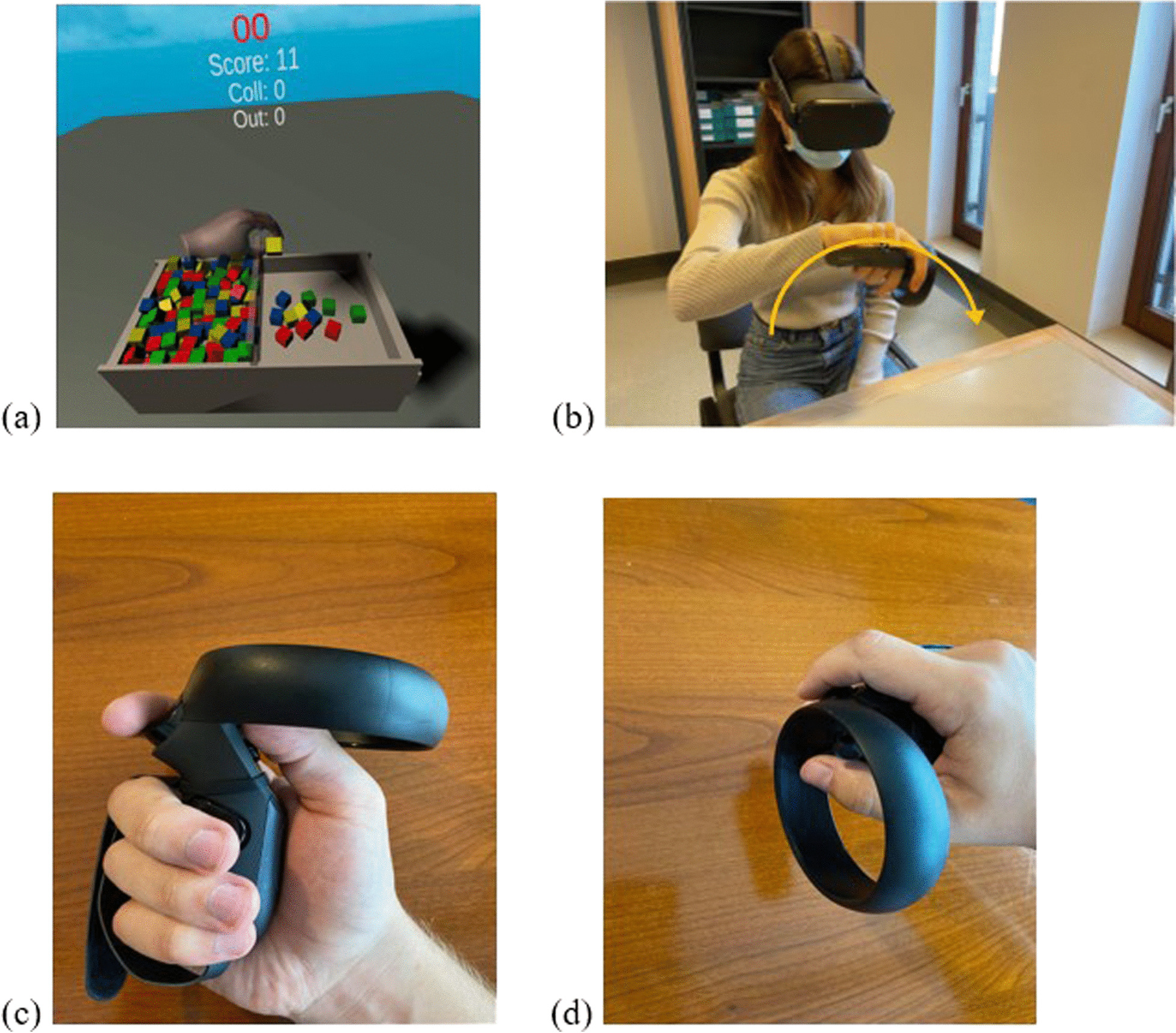
Representation of the BBT-VR (Oculus Quest). **A** The picture shows the virtual environment seen by the participant headset. It consisted of the BBT-VR, the virtual hands (which corresponded to the controllers) and the indication of the time, the score, the number of collisions with the virtual separation and the number of cubes out. **B** The picture represents a participant performing the BBT-VR with his right hand. **C** The picture shows the controller hold by a right hand. Three buttons are presented: one next to the thumb, one next to the index and one next to the middle-finger. **D** Representation of the recommended position to grab the virtual blocks in virtual reality

The BBT-VR was developed using *Unity 2019.3* software (in C# language), matching the test to the classical BBT. Participants were asked to sit on a chair, with their feet on the ground, and wearing the VR headset on their head. One controller was placed and fixed (thanks to adjustable knuckle straps) in the participants tested hand (Fig. 1a). Participants were then immersed in a neutral virtual environment in which the virtual box of the BBT was located. This box has the same dimensions as the classical box and was developed to spawn in front of the participant. Participants were asked to transfer as many virtual blocks as possible from one compartment to the other in 60 s. Participants had to reach to the cube and align their virtual hand with the cube to perform a grasping movement around the block (by pressing the buttons corresponding to the thumb-index or thumb-major or thumb-index-major grip; Fig. 1b). Once the appropriate fingers attached (entered in collision) with the virtual block, the grasping action was initiated. Then, while holding the cube (by exerting a continuous pressure on the buttons corresponding to the fingers) participants moved the cube to the other compartment of the box, taking care to pass above the median line. To complete the movement, the cube was dropped by releasing the buttons. During the test, each time the cube was picked up, the controller vibrated to give participants tactile feedback. At the end of the 60 s, an audible announcement indicated the end of the allocated time and the score was automatically recorded. Tridimensional positions and rotations of the tested hand controller were recorded (acquisition frequency = 72 Hz) and saved for kinematic analyses.

### Procedure

The experiment lasted on average 45 min and always started by giving oral explanations and instructions to participants concerning the design of the experiment. Participants were then asked to perform the classical BBT and the BBT-VR. Block randomization was used to determine the assessment order: whether the participants started with the classical BBT or started with the BBT-VR. For both tests, the dominant (less-affected) hand was evaluated before the paretic (non-dominant) hand. For the classical BBT, participants received oral instruction and were asked to train for 15 s before performing the test three times (trials of 60 s) for each hand. For the BBT-VR, the same procedure was followed except that participants could practice for one minute after receiving the test instructions. This extended period of training was set up because most participants were using VR for the first time. After each trial, participants had a short break of 30 s to avoid a potential fatigue effect.

After taking both tests, patients’ motor control was evaluated using the adaptative version of the *Upper Extremity Fugl-Meyer Assessment* (UE-FMA). This computerized and adaptative version of the UE-FMA is a stroke-specific, performance-based impairment index [24, 25]. In this version, the score is converted to a percentage thanks using a Rasch model analysis where higher percentages reveal a better upper-limb recovery.

The usability of the BBT-VR device was assessed by asking all participants to self-complete the *System Usability Scale*. The *System Usability Scale* is a quick and reliable 10 item questionnaire that measures the usability of a device [26] using a Likert scale. A score below 51% indicates awful usability, between 51 and 68% poor usability, 68% okay usability, between 68 and 80.3 good usability and above 80.3% excellent usability [27, 28].

### Kinematic data analysis

**Fig. 2:**
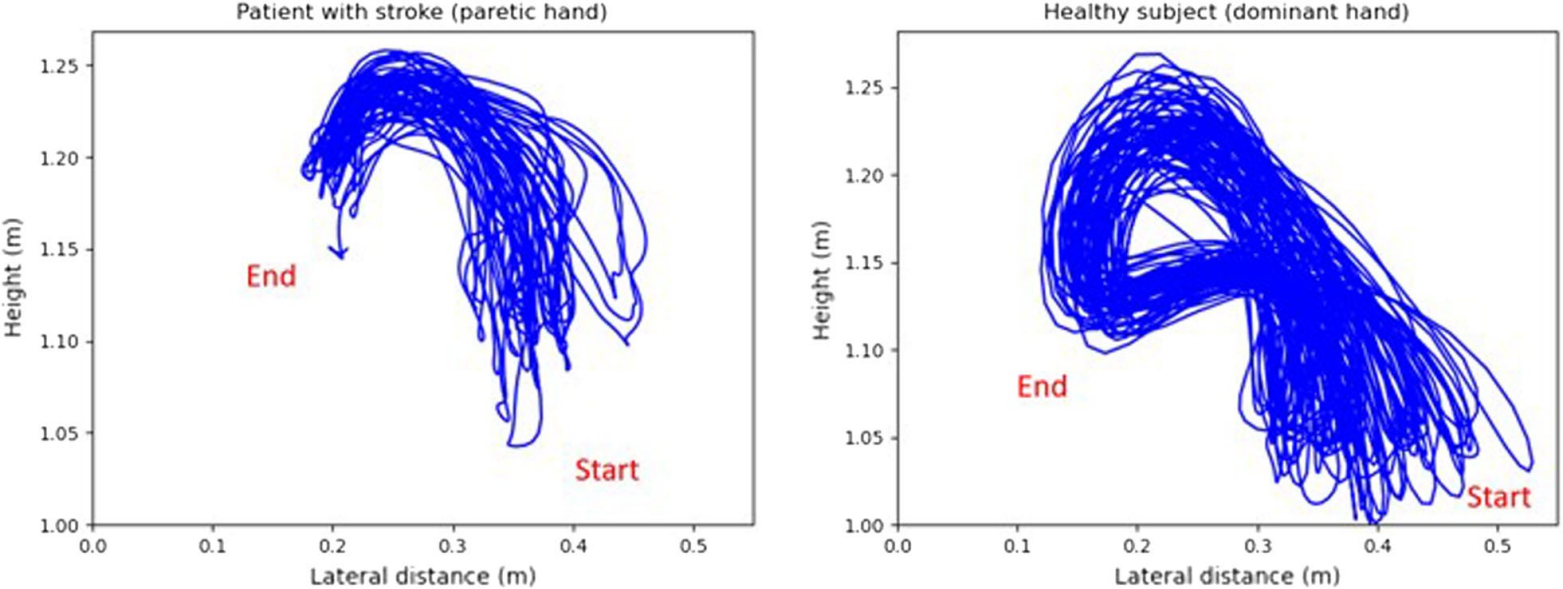
Typical graph of 3D hand movements. The blue line represents the hand position on the virtual vertical axis in relation to the virtual lateral axis. Left graph. Typical graph of a paretic hand. Right graph. Typical graph of a healthy hand

Kinematics analysis of hand position was made with a software developed in *Python*. Tridimensional hand positions first underwent a spectral analysis to distinguish the movement frequencies from noise. After analysis, a Butter-worth filter 10 Hz cut-off frequency was used to reduce noise in the signal. To assure that data was valid and reliable, a visual analysis was made by plotting hand positions in relation to time (see Fig. 2). The kinematic path was then computed by calculating the square root of the sum of each squared position. This path calculation enabled to transform tridimensional positions into dimensionless data that corresponded, in this study, to the distance covered by the hand. After that, the path that was performed during one minute was partitioned into different sub movements. A sub movement was defined as the movement performed by the hand between the grasping of a block and its successful release in the intended compartment of the virtual box. We therefore only considered the movement corresponding to the transport of a block. We did not analyze the return movement to the compartment where the subject had to grasp the next block.

For each participant’s sub movement, mean velocity, peak velocity and movement smoothness were analysed. These measures were analysed for the movement of each block during the 60 s trial and the mean measures calculated. Movement smoothness was measured with spectral arc length (SPARC) computed according to the code provided by Balasubramanian et al. [29]. SPARC is the arc length of the Fourier magnitude spectrum of the velocity signal and is independent of movement amplitude and duration [29].

### Statistical analysis

All analyses were performed with α = 0.05 using SPSS version 27.0 (IBM SPSS Statistics for Windows), SIGMAPLOT 13.0 and R. The normality of each analysis was verified using a Shapiro–Wilk test. Spearman or Pearson Correlation Coefficients and Wilcoxon Signed Rank tests or paired t-tests were computed between the scores of BTT and BBT-VR’s last trial for each hand of healthy participants and patients with stroke. Correlations were rated as small (0.1 ≤ r < 0.3), medium (0.3 ≤ r < 0.5) or large (r > 0.5) according to Cohen’s conventions [30].

The primary outcome was to assess the correlation between the number of blocks moved in BBT and BBT-VR. The sample size calculation was based on the results of previous studies and was calculated to have a correlation coefficient of 0.7 between the classical-BBT score and the BBT-VR score among each group [21, 22]. Twenty-one patients and healthy participants in each group were needed in order to achieve 95% power with a 5% significance level. The secondary outcomes were to assess the short-term test–retest reliability and usability of the BBT-VR, compute and compare kinematics among healthy participants and patients with stroke and to evaluate the effect of age on BBT and BBT-VR scores. Test–retest reliability was assessed by performing a two-way mixed model Intraclass Correlation Coefficient (ICC) between the last two trials of the BBT-VR. Reliability was rated as poor (ICC or r ≤ 0.40), moderate (0.40 < ICC or r < 0.75), or excellent (ICC or r ≥ 0.75) [31]. Minimal detectable change (MDC) was computed for each hand using the following calculation: 1.96 × Standard error of measurement × √ 2. Differences in patient’s kinematics between both hands (less-affected hand vs. paretic hand) were assessed by conducting Wilcoxon signed-rank and paired t-tests. Kinematic differences between healthy participant’s hands and the patient’s paretic hand were assessed using t-tests and Mann–Whitney tests. MDC was also computed for the kinematics of healthy participants.

## Results

Between November 2020 and March 2021, 229 patients were screened, 32 corresponded to the eligibility criteria, 27 consented to the experiment and 22 were finally enrolled. A flow chart diagram is presented in Additional file 3. Participants (patients and healthy participants) baseline characteristics are presented in Table 1. Patients were adults with stroke (5 females) with a mean age of 64 ± 10.9 years. Twelve patients suffered from a left hemispheric lesion (11 ischemic and 1 haemorrhagic) and 10 from a right hemispheric lesion (all ischemic). The majority of patients (19/22) were righthanded before their stroke. At the time of the experiment, 10 patients were in the subacute phase (7 days to 6 months since stroke onset) [32] and 12 were in the chronic phase (> 6 months since stroke onset) resulting in a median post-onset of 5.9 [1.5–16.6] months. One patient, who completed the traditional BBT, could not finish the experiment due to difficulty in understanding the functioning of the VR system and was therefore excluded from the analysis. This patient suffered from a combination of cognitive disorders (anosognosia, aphasia and apraxia). Based on their medical records, none of the patients were diagnosed with a serious cognitive impairment.

**Table 1:** Demographic data of the included participants

Participants (#)	Healthy participants (23)	Adults with stroke (22)
Age (years)	47 ± 23.9	64 ± 10.9
Gender (M/F)	10/13	17/5
Laterality (R/L)	21/2	19/3
Type of lesion (ischemic/haemorrhagic)	–	21/1
Side of lesion (R/L)	–	10/12
Time since stroke (months)	–	5.9[1.5–16.6]
FMA (%)	–	76.3 ± 21.2
BBT (dominant; less-affected hand) (#blocks)	75.7 ± 12.72	56 [52–66]
BBT (non-dominant; paretic hand) (#blocks)	72.8 ± 12.37	36.8 ± 19.96
# Healthcare professionals	12	/

*FMA* Upper Extremity Fugl-Meyer Assessment, *M* male, *F* female

Twenty-three healthy participants (13 females) with a mean age of 47 ± 23.9 years also participated in the experiment. Among them, 12 (52%) worked as healthcare professionals and a large majority (21/23) were righthanded.

### Primary outcome—concurrent validity

**Fig. 3:**
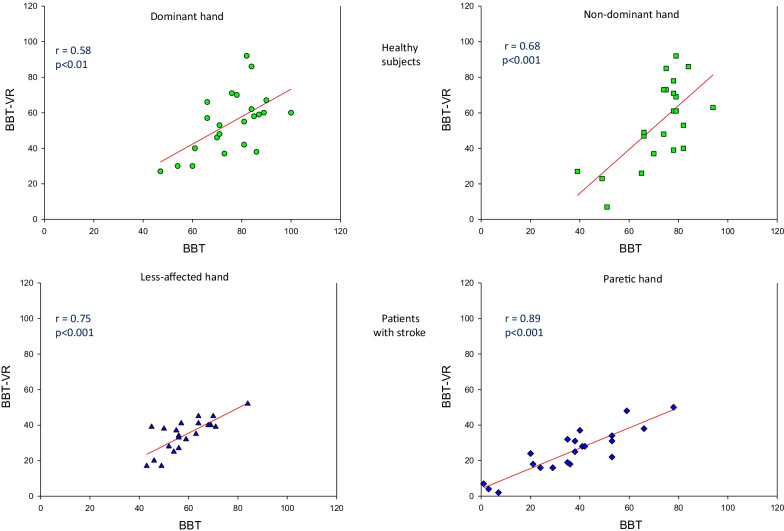
Correlation between the classical BBT score and BBT-VR score. Each point represents the score obtained by each participant’s hand during the third trial of the BBT-VR in relation to the score obtained during the third trial of the BBT. Pearson correlation coefficients (r) and their p-value (p) are presented at the left side of each graph. Linear regressions are plotted for each graph (in red). Scores of healthy participants are presented in green (dominant hand = circle; non-dominant hand = square) and scores of patients with stroke in blue (less-affected hand = triangle; paretic hand = diamond)

Correlations between the classical BBT and BBT-VR scores were large and significant for all comparisons: for patients (paretic hand, r = 0.88; p < 0.001; less-affected hand, r = 0.75; p < 0.001) and for healthy participants (non-dominant hand, r = 0.68; p < 0.001; dominant hand r = 0.58; p < 0.001) (Fig. 3). These correlations demonstrated the concurrent validity of the BBT-VR. On the whole, participants moved between 30 and 40% less blocks in virtual reality (42.9 ± 20.63) than in the classical test (61.6 ± 20.78).

### Secondary outcomes

Results of test–retest reliability are presented in Table 2. A significant difference was found between the BBT-VR score of the first and second trials for all participants. However, no differences were found between the second and third trials. Consequently, the first trial was removed from the correlation analysis and considered as an additional training. Regarding adults with stroke, strong correlations were shown between the second and third trial of the BBT-VR for both paretic (ICC = 0.87; p < 0.001) and less affected hands (ICC = 0.84; p < 0.001). MDC was of 5.2 for paretic hand and 4.2 for less-affected hand. Regarding healthy participants, strong correlations were also observed between the second and third attempt of the BBT-VR for both dominant (ICC = 0.89; p < 0.001) and non-dominant hands (ICC = 0.94; p < 0.001). MDC was of 6.7 for dominant and 8.4 for non-dominant hand.

**Table 2:** Intraclass correlation coefficient between second and third trial of the BBT-VR

	Trial 1	Trial 2	Trial 3	ICC (2vs3)	p-value (Anova)
Healthy participants					
Dominant hand	44.3 ± 16.20	51 ± 16.09†	54.5 ± 17.02	0.89*	< 0.001
Non dominant hand	47.8 ± 18.23	53.7 ± 19.28†	55.2 ± 22.3	0.94*	< 0.001
Patients with stroke					
Less affected hand	24.5 ± 9.06	31.9 ± 10.31^†^	34.5 ± 9.36	0.84*	< 0.001
Paretic hand	17 [14–29]	18 [13.5–30]†	25 [17–33]	0.87*	0.04

ICC = Intraclass correlation coefficient; 2 = Trial 2; 3 = Trial 3; results are presented as means ± standard deviation or median [quartile 1–quartile 3]; * = significant p-value regarding ICC; ^†^ = significant p-value between trial 1 and 2 (post-hoc); ^‡^ = significant p-value between trial 2 and 3 (post-hoc)

Mean usability score (according to the *System Usability Scale*) was of 79 ± 13.0% for patients with stroke and 78 ± 11.7% for healthy participants which results in a good (almost excellent) usability rating.

**Fig. 4:**
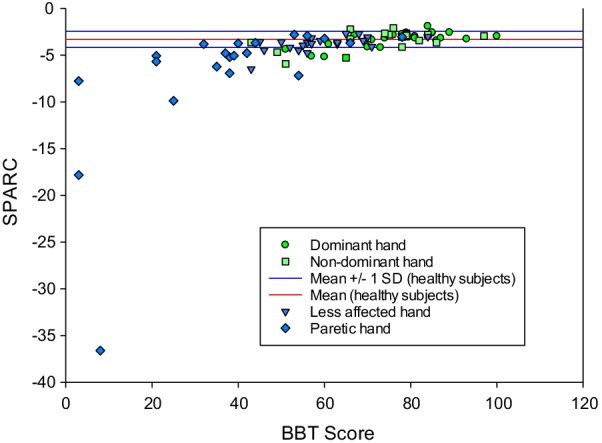
Scatter plots representing kinematic measures of participants in relation to their BBT score. Each point represents the SPARC smoothness unit obtained by each participant’s hand during the best trial of the classical BBT-VR in relation to the score obtained during the best trial of the BBT. The red lines represent the mean SPARC obtained among healthy participants ± 1 standard deviation

Norms resulting from healthy participants were of 0.22 ± 0.086 ms^−1^ for V_mean_, 0.96 ± 0.341 ms^−1^ for V_peak_ and − 3.31 ± 0.862 for the SPARC (Fig. 4). MDC was of 0.035 ms^−1^ for V_mean_, 0.139 ms^−1^ for V_peak_ and 0.352 ms^−1^ for the SPARC.

Among patients with stroke, mean velocity, peak velocity and smoothness were found to be significantly different between the paretic hand (V_mean_ = 0.12 ± 0.052 ms^−1^; V_peak_ = 0.60 ± 0.202 ms^−1^; SPARC = − 5.04[− 7.050 to − 3.682]) and the less-affected hand (V_mean_ = 0.17 ± 0.063 ms^−1^; V_peak_ = 0.813 ± 0.311 ms^−1^; SPARC = − 3.63[− 4.136 to − 3.310]). All kinematic measures were significantly different between healthy participants’ hands and paretic hands of patients with stroke.

## Discussion

This study aimed to develop and validate a virtual immersive version of the BBT to assess manual dexterity among patients with stroke and healthy participants. Strong correlations between BBT and BBT-VR scores demonstrated concurrent validity even though there was a significant score difference between test versions (20–30% of blocks). Test–retest reliability was found to be excellent, and usability was rated as good almost excellent. The BBT-VR provides quantitative and objective kinematic data differentiating patients with stroke from healthy participants.

### Findings of previous studies

These results can be compared to the study of Ona et al. (2020) which evaluated the concurrent validity of another virtual BBT among patients with Parkinson Disease [22]. Pearson correlations obtained between the scores from the virtual BBT and the classical BBT were slightly inferior than in the present study (r = 0.49 for the affected hand and r = 0.51 for the less-affected hand). This difference could be attributed to the lack of tactile feedback provided in their version of the virtual BBT.

Previous studies demonstrated excellent inter-rater and test–retest reliability of the classical BBT when assessed among healthy participants, and patients with paresis and spasticity [7–10]. These results are in line with our study since test–retest reliability of the virtual BBT was found to be excellent for all participants. However, the correlations of the virtual BBT were assessed in a short-term period between the last two attempts and were slightly inferior to those of the classical BBT. The virtual BBT reported by Ona et al. (2020) also demonstrated an excellent reliability among patients with Parkinson’s Disease [22].

Participants rated the present BBT-VR usability as very good. In the Oña study, virtual BBT test usability was measured by delivering a satisfaction questionnaire to Parkinson's disease specialists and patients [22]. Both groups showed a good to excellent level of satisfaction. However, in Gieser’s study, questions were asked to find out the preference of use between the virtual BBT and the BBT [23]. Participants showed a preference in favour of the BBT and revealed difficulty in grabbing the virtual blocks. These results suggest that virtual assessments are not all easy to use and well accepted by potential users. Moreover, these virtual assessments may require a training period as showed in our study.

Lastly, this study aimed to compute and compare kinematic data among healthy participants and patients with stroke. The literature advocates using kinematics to enable a better understanding of recovery and the underlying mechanisms of functional improvements after a stroke [33, 34]. In our study, kinematics were used to objectively quantify patients’ movement quality. Kinematics of interest were hand movement speed (V_mean_, V_peak_) and smoothness (SPARC). In 2012, a study showed that healthy participants had a superior smoothness index than patients with stroke when performing a reaching task through an end-effector robotic device [35]. In 2016, a research paper that developed a non-immersive virtual version of the UE-FMA revealed that upper-limb movement smoothness of patients with stroke was significantly better on the less-affected side than the paretic side [36]. Although these findings are in line with our results, smoothness outcomes were different from our study since they used speed ratio or a logarithmic dimensionless jerk motion analysis instead of a SPARC index (which is independent of the movement amplitude and duration). Another study by Ona et al., demonstrated the feasibility of computing a SPARC index among patients with stroke when performing the UE-FMA [37].

### Virtual environment vs. reality

The number of blocks displaced in the BBT was significantly superior to the number of blocks displaced in BBT-VR for each hand of all participants. This score difference is in line with previous research that underlined the existence of a gap between physical and virtual assessments [21–23]. These findings may be explained by different factors.

First, the fact that VR systems do not provide realistic tactile feedback may lead to a decreased performance. Indeed, several studies have shown that providing haptic and tactile feedback had a positive influence on performance when achieving functional tasks [38, 39]. However, up to now, the device used in VR (such as the *Leap Motion*) did not supply any tactile feedback. Using controllers may help to deliver haptic response such as vibrations although they do not fully correspond to tactile sensory stimulation. To address this issue, it could be interesting to develop the assessments with haptic gloves or with real objects in mixed reality.

Second, it is known that the performance in virtual tasks is influenced by experience with videogames [40]. Individuals with a longer history of videogaming tend to obtain better results when performing a virtual task. Cho et al. demonstrated a positive strong correlation between the BBT-VR score and the level of video game/VR experience [21]. Moreover, elderly people (> 65 years) represent less than 2% of the video-gamers and mean age of patients with stroke included in our study was of 63.5 years [40]. Hence, it could be hypothesized that this score difference between BBT and BBT-VR would be reduced in a younger population.

Lastly, the hand grip (including hand opening and closing) may appear unnatural to some participants which could have lead to an increased learning and adaptation period responsible for the score difference. However, the BBT-VR seems to have a great content validity as the position of the different controller buttons to be pressed allowed participants to grab and displace the blocks with a realistic thumb-index/thumb-major/global pinch. Moreover, the size of the controller (3 cm) was close to the size of the BBT blocks (2.5 cm).

### Implications for clinical practice

As a result of this study, several perspectives can be put forward. First, the BBT-VR offers patients the possibility to evaluate their manual dexterity independently in hospitals, in rehabilitation centres or at home. Moreover, the price of the device (± 350€/415US$) is affordable, matching that of the BBT (± 300€/356US$) and it does not require a computer. This self-evaluation may therefore increase the frequency of assessments and responds to the recommendation of performing regular monitoring and evaluations with less intervention from the evaluator [4]. Similarly, implementing this manual dexterity self-assessment at the patients’ home could help clinicians to assess the patient on the long term and detect a potential drop in performance with the transition from hospital to home.

Second, the BBT-VR enables to objectively measure performance by computing and analysing hand kinematics aside from a score of manual dexterity. This information will help clinicians to understand whether patients continue to improve while their BBT score has reached a plateau.

Lastly, it would be interesting to implement the BBT-VR in a more complete VR treatment protocol that comprises assessments and serious games. With this VR protocol, patients could, on one side, self-rehabilitate through VR games especially designed to work on motor and cognitive impairments and, on the other side, self-evaluate through motor and cognitive VR-based assessments. This would help patients to get used to VR and see whether playing serious games help them to improve their motor and cognitive performances.

### Limitations

The BBT-VR has several limitations. First, the movement to perform with the controllers to pick up the virtual blocks may be slightly different from natural grasping since the controller’s button positions are fixed. Second, it could be interesting to track participants elbow, arm, shoulder and trunk to detect any compensations during the test, but this VR system can only track hand position. This could be performed by implementing trackers on the body parts of interest and recording their position in real time. Lastly, the BBT-VR may not suit all patients suffering from important cognitive disorders such as anosognosia, fluent aphasia or apraxia. It could also be interesting to compare the ability of these patients to perform the *BBT* vs *BBT-VR*.

This study also has limitation. The test–retest reliability was assessed through the same session whereas the recommended time-interval between testing is around two weeks.

## Conclusion

This study showed that the BBT-VR is a valid, short-term reliable, and usable tool to assess manual dexterity among patients with stroke and healthy participants. The BBT-VR provides quantitative and objective kinematic data differentiating patients with stroke from healthy participants.

## Supplementary Information

**Additional file 1. **Raw data. Data supporting the results and conclusion of this paper.

**Additional file 2.** Movie representing the BBT-VR. Video showing on the right side, someone performing the BBT-VR with the headset and controllers and on the left side, what this person could see through the headset device.

**Additional file 3. **Flow chart diagram of the included patients. Flow chart diagram representing the recruitment and inclusion process of patients with stroke.

## Data Availability

The dataset supporting the conclusions of this article is included within the article (and its additional file(s)).

## Abbreviations

BBT: *Box and block test*
BBT-VR: Immersive virtual reality-based version of the *Box and block test*
VR: Virtual reality
UE-FMA: *Upper extremity Fugl-Meyer assessment*
Vmean: Mean velocity
Vpeak: Peak velocity
SPARC: Spectral arc length
MDC: Minimal detectable change
