# Supplementary figures and images for: Concurrent validity of an immersive virtual reality version of the Box and Block Test to assess manual dexterity among patients with stroke
Source: J Neuroeng Rehabil. 2022 Jan 22;19:7. doi: 10.1186/s12984-022-00981-0 (PMC8783988; doi:10.1186/s12984-022-00981-0)

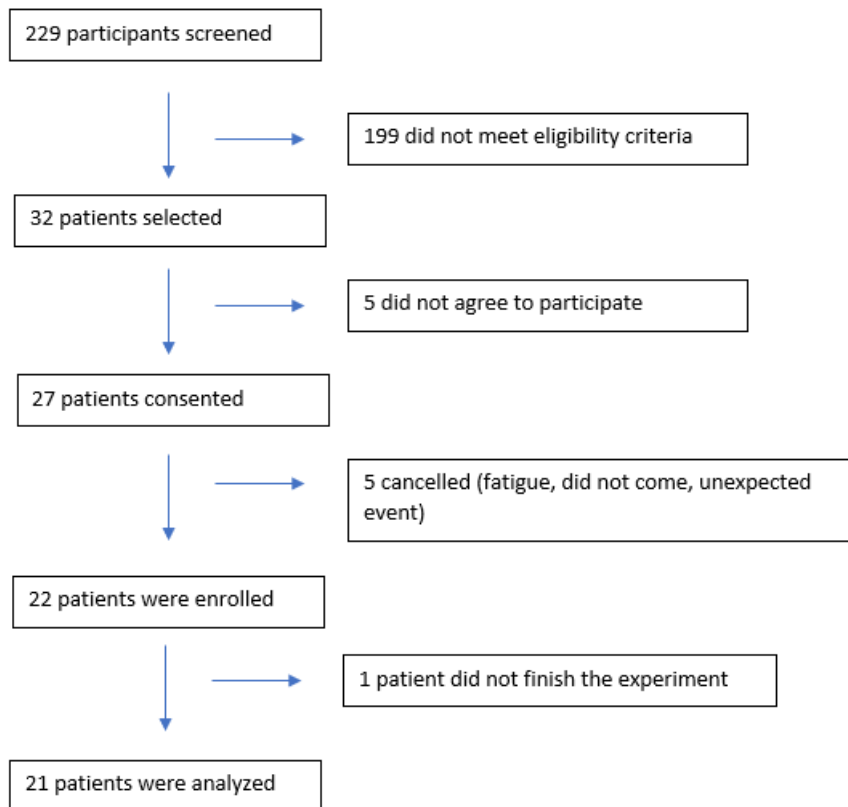

Supplement: Supplementary file 3 — Additional file 3. Flow chart diagram of the included patients. Flow chart diagram representing the recruitment and inclusion process of patients with stroke. [file 12984_2022_981_MOESM3_ESM.pdf]
